# Mouth Washes

**Published:** 1890-02

**Authors:** 


MOUTH WASHES.—Hygiene of the teeth is as important as that of other parts of the body; and prophylaxis in this direction will prevent decay as well as keep the teeth clean and the breath sweet. Monte, in the *Deutsche med. Wochenschrift*, October 31, 1889, gives the following two formulas for prophylactic mouth washes:

| | | |
|---|---|---|
| ℞ | Acidi borics, | gr. xxxviij. |
| | Aquæ destillatæ, | f ℥ vij. |
| | Tincturæ myrrhæ, | ♏ xxxxviij. M. |
| ℞ | Sodii salicyl. | gr. xxxxiij. |
| | Aquæ destillatæ, | f ℥ vij. |
| | Tincturæ myrrhæ, | ♏ xxxxviij. M. |

Sig. Wash out the mouth several times daily with either of the above formulæ.—*Med. and Surg. Reporter.*

